# Retraction: Assessment of photocatalytic potentiality and determination of ecotoxicity (using plant model for better environmental applicability) of synthesized copper, copper oxide and copper-doped zinc oxide nanoparticles

**DOI:** 10.1371/journal.pone.0215711

**Published:** 2019-04-16

**Authors:** The PLOS ONE Editors

Following publication, concerns were raised about the accuracy of the data reported in this article [1]. The *PLOS ONE* Editors have discussed the concerns with the authors and have considered the information and data provided, along with input from members of the Editorial Board. A number of issues could not be fully resolved, and the outstanding concerns are as follows:

- The chromatogram in Fig 6e is incorrect and belongs to a different compound. The Y axis of this chromatogram was modified from the original file with a conversion from mV to counts and a reduction in the peak intensity. The corresponding author has stated that there was a mistake when collecting the data files from the shared repository of the HPLC machine. A corrected replacement chromatogram was provided. The corresponding author has stated that the Y axis was modified from the original to reduce instrumental artefacts.
- The elution times on the ESI-MS plots in Fig 6f-i are related to the peaks shown in the incorrect Fig 6e chromatogram, rather than the peaks shown in the corrected chromatogram image. The corresponding author has stated that fractions were collected at different elution times and analysed on a separate instrument for ESI-MS and that the incorrect elution times were assigned to the fractions analysed by ESI-MS during figure preparation.
- The corresponding author has clarified that the structures in Figs 6f-i represent protonated species of the same molecule. The molecular weight reported for the abundant compound at the specific elution time in Fig 6g does not correspond to the structure presented.
- In Fig 14 different regions are defined in panel a compared to panels b-e, and panel a lacks a region defining apoptotic events; the source of the data for the control column of the bar chart in panel f is therefore unclear. Conclusions drawn from comparisons made between inconsistently defined regions are not fully supported. It is not clearly indicated how replicate experiments were used to generate the data for panel f, nor is there an indication of experimental errors observed.
- The Results text describes dosage effects on both cell cycle dynamics and apoptosis, but no data across different dosages is presented to support these findings.
- The corresponding author has stated that FACS data was provided by the Central Instrument Facility, Bose Institute, Kolkata. The original scatter plot, histogram, and statistics output files were provided; however, the raw .fcs files are not available. Discrepancies were noted between some of the scatter plots and histograms in the figures when compared to these original output files. The corresponding author has indicated that in Fig 14a and 14c the scatter plot and inset histogram plots in each panel are derived from two different samples from the same group. The corresponding author has also stated that in some of the histogram plots, the images were edited using image processing software to remove the intensified peaks for exclusion of signals generated due to debris.

In light of the above issues, the *PLOS ONE* Editors retract this article owing to concerns about the preparation of images and the reliability of the presented data which compromise the overall validity of the findings.

DD, AKD, DVK, BG, AP, SG, and AM did not agree with retraction.
